# Association of Intratumoral Microbiota Modulation with Prostate Cancer Progression: A Microbiome Analysis of Prostatic Tissue

**DOI:** 10.3390/biomedicines13081929

**Published:** 2025-08-07

**Authors:** Jae Heon Kim, Hoonhee Seo, Sukyung Kim, Md Abdur Rahim, Sujin Jo, Indrajeet Barman, Hanieh Tajdozian, Faezeh Sarafraz, Md Sarower Hossen Shuvo, Ho-Yeon Song, Yun Seob Song

**Affiliations:** 1 Department of Urology, School of Medicine, Soonchunhyang University, Seoul 14584, Republic of Korea; 2 Human Microbiome Medical Research Center (HM∙MRC), School of Medicine, Soonchunhyang University, Chungnam 31538, Republic of Korea; 3 Department of Microbiology and Immunology, School of Medicine, Soonchunhyang University, Chungnam 31151, Republic of Korea

**Keywords:** prostate cancer, intratumoral microbiome, 16S rRNA amplicon-based profiling, cancer progression, microbiome-targeted therapy

## Abstract

**Background:** The involvement of the intratumoral microbiome in prostate cancer progression is becoming increasingly acknowledged. This study analyzed the microbiome of prostate cancer tissues from patients with localized prostate cancer (LPC, stages 1–2) and advanced prostate cancer (APC, stages 3–4) to determine its association with cancer progression. **Methods:** Paraffin-embedded tissue samples obtained during radical prostatectomy underwent 16S rRNA amplicon-based profiling. **Results:** The profile of the bacterial communities in LPC and APC differed remarkably. While species diversity remained stable, species richness (as determined by the ACE analysis) was significantly lower in APC, correlating with a decrease in *Enhydrobacter* (which is more abundant in LPC) and an increase in *Lautropia* (enriched in APC). The role of *Lautropia* in the progression of cancer was confirmed by in vitro studies employing cell lines from prostate cancer. **Conclusions:** These findings demonstrate the potential of microbiome-targeted interventions in the management of prostate cancer.

## 1. Introduction

The human microbiome refers to the complex ecology of bacteria, fungi, viruses, and archaea that inhabit the human body. It is essential for maintaining health and homeostasis. This microbial community has a profound influence on various physiological processes, including immune system development and regulation, metabolic function, and protection against pathogens [1,2]. Increasingly, studies are highlighting the importance of the microbiome in the pathogenesis and progression of numerous diseases, particularly cancers [3].

The concept of the “tumor microbiome”, encompassing the microbial communities residing within and surrounding tumors, has emerged as a significant area of investigation [4]. This intratumoral microbiome interacts dynamically with the tumor microenvironment, influencing key cancer hallmarks, including inflammation, immune evasion, angiogenesis, metastasis, and response to therapy [5,6]. The composition of the tumor microbiome varies across different cancer types and disease stages, with significant implications for tumor progression and patient outcomes [7,8].

The relationship between the microbiota and disease progression in prostate cancer is becoming more widely acknowledged, although the mechanisms remain largely unexplored [9]. While traditionally considered a relatively sterile organ, the prostate harbors a complex and dynamic microbiota that may influence the local microenvironment [10]. According to recent research, prostate tumors have distinct microbial signatures compared to benign tissue, with malignant lesions exhibiting an enrichment of bacterial taxa, including *Propionibacterium*, *Streptococcus*, and *Escherichia* [11,12]. Furthermore, persistent prostatic inflammation is a risk factor for the advancement of cancer. It has been connected to dysbiosis in the gut and urine microbiomes [13]. Nevertheless, some research indicates that aggressive tumors have less microbial diversity [14], while other studies suggest that certain infections, such as *Fusobacterium*, may encourage metastasis through inflammatory pathways [15]. Alterations in the abundance and composition of this community are linked to inflammation, progression to advanced disease, and potentially, metastasis [16,17]. However, the extent to which this is genuinely the case and the underlying mechanisms require further investigation.

The tumor microenvironment, influenced by the microbiome, significantly shapes the cancer cell phenotype, promoting proliferation, invasion, and metastasis [13]. This microenvironment contains a complex mix of cells, including immune cells, fibroblasts, endothelial cells, and cancer-associated fibroblasts (CAFs). Additionally, it includes signaling molecules, extracellular matrix components, and metabolites produced by the tumor and surrounding tissue [7,14]. Microbial-derived metabolites have been shown to influence several aspects of the tumor microenvironment, impacting inflammation, immune function, and angiogenesis [6,15]. For example, short-chain fatty acids (SCFAs) from gut bacteria can influence immune responses by modulating the activity of immune cells, such as cytotoxic T lymphocytes (CTLs) and regulatory T cells (Tregs) [8,16]. Furthermore, lipopolysaccharides (LPSs) produced by Gram-negative bacteria can promote inflammation and contribute to the development of resistance to cancer therapies [9,10,17].

Recent advances in microbiome research have opened new avenues for precision oncology, where microbial profiling may complement genomic and transcriptomic data to refine cancer diagnostics and prognostics [18]. Microbial signatures could serve as non-invasive biomarkers for early detection, risk stratification, and monitoring of therapeutic response [18]. Moreover, modulating the microbiome through probiotics or fecal microbiota transplantation (FMT) presents a promising adjunct to conventional cancer therapies [19,20]. Hence, understanding the interplay between microbial communities and host cellular pathways may unlock novel therapeutic targets, especially in cancers like prostate cancer.

The influence of specific bacterial taxa on prostate cancer progression has only begun to be elucidated. Studies have implicated specific bacterial genera in promoting tumor growth, invasion, and metastasis [21].

However, a better comprehension of the complex mechanisms by which these bacteria influence tumor biology is crucial for the development of effective therapeutic strategies. This study utilizes next-generation sequencing (NGS) to investigate the relation between alterations in the intratumoral microbiome and prostate cancer progression from localized to advanced stages, aiming to identify potential microbial biomarkers predictive of disease severity.

## 2. Materials and Methods

### 2.1. Subject Recruitment and Sample Collection

The study enrolled 26 patients from Soonchunhyang University Hospital’s Urology Department who had been diagnosed with prostate cancer, including subjects from an earlier study by the same research team [22]. The participants had no significant underlying conditions, had not undergone prior treatment, and had not recently used antimicrobials. All underwent radical prostatectomy, with paraffin tissues collected during surgery. Participants were split into two groups: one for localized cancer (stage 1–2 prostate cancer patients) and another for advanced cancer (stage 3–4 patients). Table S1 provides specifics on the patients’ clinical attributes. This study was conducted in accordance with the guidelines of the Declaration of Helsinki [23]. This protocol was approved by the Local Scientific Ethics Committees of Seoul hospital (2017-02-002) on 8 February 2017, Bucheon hospital (2017-03-004) on 25 May 2017, Cheonan hospital (2017-03-031-024) on 5 April 2017, and Gumi hospital (2017-03-031-002) on 20 February 2017.

### 2.2. 16S rRNA Amplicon-Based Profiling of Prostate Cancer Tissues

This study reanalyzed previously generated and published 16S rRNA sequencing data [22], with a specific focus on investigating the association between intratumoral microbiota modulation and prostate cancer progression. It therefore provides a concise description of the methods. The formalin-fixed, paraffin-embedded (FFPE) prostate tissue samples were used to extract genomic DNA and analyze the intratumoral microbiome. The quality and quantity were checked following our previously published study [24]. Afterward, the genomic library was prepared following our published protocol, and finally, sequencing was conducted [24]. Detailed sequencing metrics, including read counts, quality filtering results, and ASV statistics, were previously reported in our earlier publication [22]. Trimmomatic [25] was used to pre-process the raw reads, removing adapters and low-quality bases to create clean reads. Myers & Miller’s alignment technique [26] was then used to trim the primers at a similarity cutoff of 0.8. Using hmm profiles, nhmmer identified non-specific amplicons that do not encode 16S rRNA in the HMMER software program [27]. The derep_fulllength command in VSEARCH2 was used to cluster redundant reads and extract unique reads [28]. VSEARCH’s usearch_global command [28] was used for taxonomic assignment, and Myers & Miller’s technique [26] was used for more accurate pairwise alignment. FLASH software was used to compile paired-end readings (version 1.2.11), and sequence processing was conducted using the QIIME pipeline [29,30]. The RDP classifier was used to classify operational taxonomic units (OTUs), which were then mapped to the human microbiome database using a Bayesian approach with a 97% cutoff [31]. Rarefaction curves were computed using an online program (www2.biology.ualberta.ca/jbrzusto/rarefact.php) (accessed on 24 January 2023). The components of the bacterial populations were identified using ANOSIM, PLS-DA, and nonparametric analysis of Adonis distance matrices. Bacterial community diversity and richness were assessed using alpha-diversity indices, including Chao 1, ACE, Simpson, Shannon, and Good’s coverage. Statistical differences in alpha diversity were assessed using the Wilcoxon rank-sum test, while distinctions between bacterial groups were assessed using PERMANOVA (Permutational Multivariate Analysis of Variance) based on four dissimilarity indices (Jensen–Shannon, UniFrac, weighted UniFrac, and Bray–Curtis) and visualized using principal component analysis (PCA) [31,32,33,34]. Taxonomic differences between groups were identified using LEfSE (version 1.1.01).

### 2.3. Evaluation of Taxonomic Biomarker Strain in Prostate Cancer Cell Lines

The impact of *Lautropia dentalis*, a key biomarker in cancer progression, on prostate cancer cell viability was evaluated using an MTT assay [35]. DU-145 prostate cancer cells (KCLB 30081) were provided by the Korean Cell Line Bank (KCLB, Republic of Korea). The cells were cultured as described in our previous study [24]. The *L. dentalis* strain (NCCP CO19) was obtained from Chosun University (Republic of Korea) and cultured anaerobically in trypticase soy broth (TSB) (Kisan Bio, Republic of Korea) at 37 °C for 72 h. For this assay, DU-145 cells were seeded and treated with *L. dentalis*. They were then incubated for 6 or 18 h. Following treatment, the MTT solution was added, followed by incubation, and finally, absorbance was assessed at 570 nm by a VICTOR ^®^ Nivo™ Multimode Microplate Reader (PerkinElmer, USA) [24,36]. All data are presented as mean ± standard deviation (SD). Statistical differences were identified by unpaired Student’s *t*-test following confirmation of normality and homogeneity of variances.

## 3. Results

### 3.1. Patient Characteristics

This study included 26 patients (median age 72.5 years; range 58–82 years) diagnosed with prostate cancer who underwent radical prostatectomy. The cohort comprised 21 patients with localized prostate cancer (LPC; stages 1–2) and 5 patients with advanced prostate cancer (APC; stages 3–4), as determined by pre-operative imaging and pathological examination. The mean serum prostate-specific antigen (PSA) level before surgery was significantly higher in the APC group compared to the LPC group (*p* = 0.003, Mann–Whitney U test). Gleason scores and pathological T stages were also significantly higher in the APC group (*p* < 0.05 for both, Mann–Whitney U test). Detailed clinical and pathological characteristics are presented in Table S1.

### 3.2. Microbial Community Composition and Diversity

**Figure 1:**
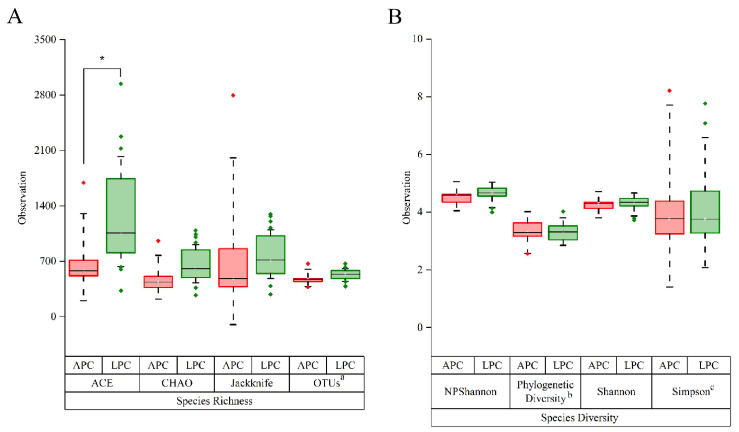
Alpha-diversity analysis in LPC and APC groups. (**A**) Boxplot comparison of species richness metrics (ACE, CHAO, Jackknife, OTUs) between LPC (green) and APC (red) groups. A remarkable increase in species richness was observed in the LPC group, as indicated by the ACE index (* *p* < 0.05). (**B**) Boxplot comparison of species diversity metrics (NPShannon, Phylogenetic Diversity, Shannon, Simpson) between LPC and APC groups, showing no significant differences in overall species diversity. The boxplot’s edges indicate the first and third quartiles, and the median value is shown horizontally by the thick block band. Statistical significance was examined using the Wilcoxon rank-sum test. ^a^ the OUTs values were multiplied by 2; ^b^ Phylogenetic Diversity values were divided by 10; and ^c^ Simpson values were multiplied by 100.

Alpha-diversity analysis, using several metrics (Shannon, Simpson, Chao1, ACE), revealed no significant difference in overall microbial diversity between LPC and APC (*p* > 0.05 for all metrics, Mann–Whitney U test). However, according to ACE analysis, the species richness in the APC group was significantly lower than that in the LPC group (*p* = 0.02, Mann–Whitney U test) (Figure 1).

**Figure 2:**
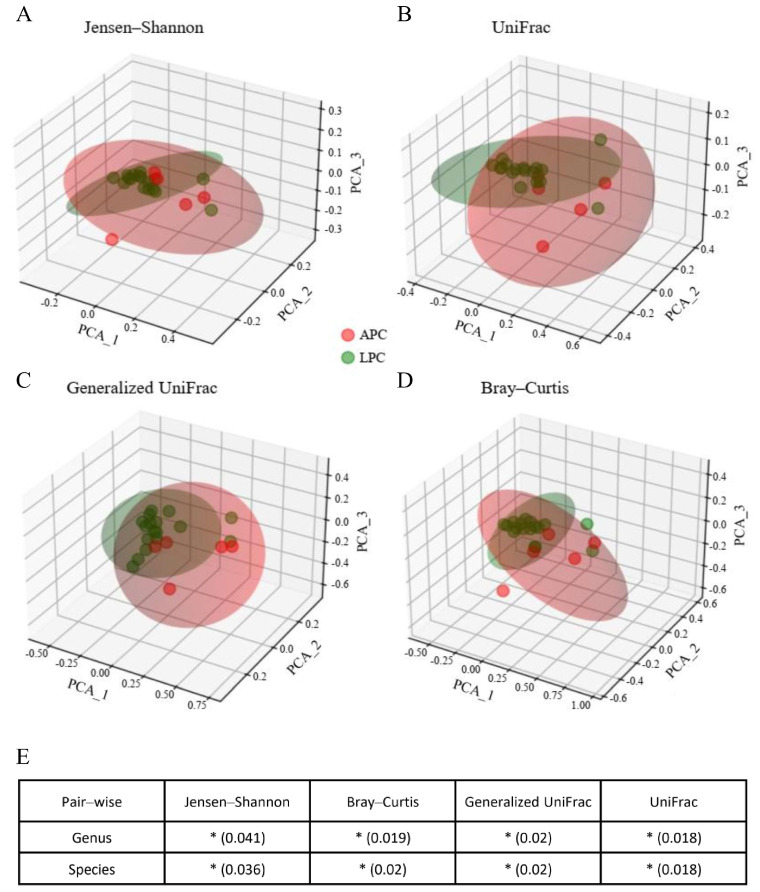
Principal coordinate analysis (PCoA) in LPC and APC groups. (**A**) A PCoA plot based on the Jensen–Shannon index reveals distinct clustering between the LPC and APC groups. (**B**) UniFrac-based PCoA reveals phylogenetic differences between the two groups. (**C**) Generalized UniFrac PCoA highlights microbial divergence considering abundance-weighted variations. (**D**) Bray–Curtis dissimilarity analysis shows compositional differences in bacterial communities. (**E**) PERMANOVA analysis confirms statistically significant microbial differences across all metrics at the genus and species levels (* *p* < 0.05). The three-dimensional plots visualize similarities between microbial communities across samples. The axes labeled PCA_1, PCA_2, and PCA_3 refer to the first three principal components derived from principal coordinate analysis.

Beta-diversity analysis, assessing the overall dissimilarity in microbial community composition between groups, was carried out utilizing principal coordinate analysis (PCoA) according to four dissimilarity indices (Jensen–Shannon, UniFrac, weighted UniFrac, and Bray–Curtis). The PCoA plots (Figure 2) separate the LPC and APC groups across all four indices. PERMANOVA analysis revealed a significant difference in microbial community composition (*p* < 0.001 for all indices). This significant difference persisted even after accounting for clinical variables, including age, PSA level, and Gleason score.

### 3.3. Taxonomic Differences

**Figure 3:**
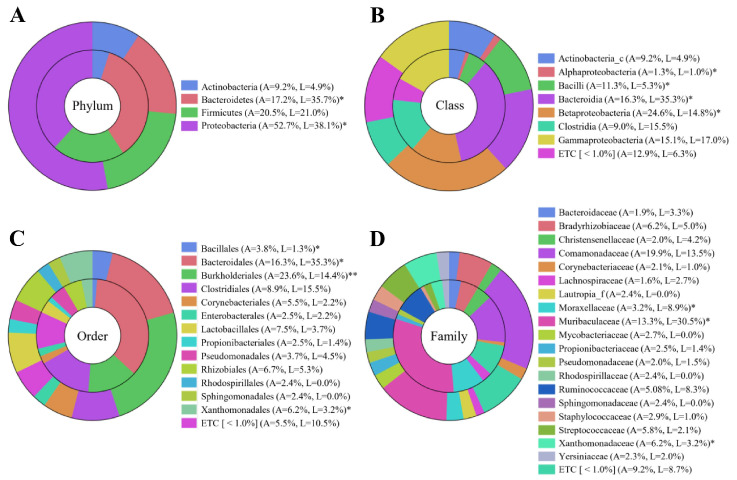
Taxonomic composition of bacterial communities in LPC and APC groups. (**A**) A phylum-level comparison reveals a higher abundance of Proteobacteria in APC (52.7%) and Bacteroidetes in LPC (38.1%) (*p* < 0.05), while Firmicutes and Actinobacteria remain relatively stable. Additionally, Bacteroidetes showed notable significance, accounting for 17.2% in APC and 35.7% in LPC. (**B**) Class-level analysis reveals a significant upregulation of Bacteroidia in the LPC group, whereas Alphaproteobacteria, Bacilli, and Betaproteobacteria were significantly increased in the APC group (*p* < 0.05). (**C**) Order-level differences include a higher prevalence of Bacteroidales in LPC (*p* < 0.05), while Burkholderiales are enriched in APC (*p* < 0.01). (**D**) A comparison of the taxonomic family levels showed that Moraxellaceae and Muribaculaceae were significantly more abundant in LPC than in APC. The Wilcoxon rank-sum test was utilized to analyze the statistical significance (* *p* < 0.05; ** *p* < 0.01). ETC represented a relative abundance of less than 1%. The inner ring represents the LPC group, while the outer ring represents the APC group.

Analysis of taxonomic composition at different levels (phylum, class, order, family) revealed significant differences between LPC and APC (Figure 3). At the phylum level, Proteobacteria was remarkably more abundant in the APC group (52.7%) compared to the LPC group (38.1%), while Bacteroidetes showed the opposite trend (LPC 35.7%, APC 17.2%) (*p* < 0.05). These trends were consistent across several lower taxonomic levels (Table S2).

### 3.4. Identification of Biomarker Taxa

**Figure 4:**
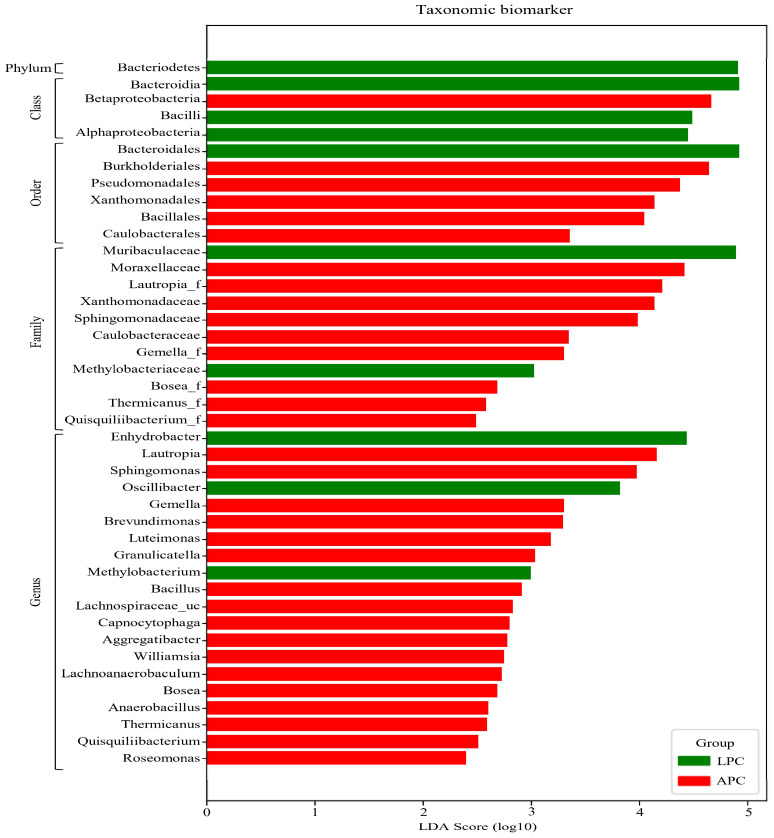
Taxonomic biomarker analysis of bacterial communities in LPC and APC using linear discriminant analysis effect size (LEfSe). An LEfSe analysis was employed to identify taxonomic biomarkers (phylum, class, order, family, and genus) differentiating LPC and APC groups, with taxa showing a linear discriminant analysis (LDA) score greater than 4 considered key biomarkers. At the genus level, *Enhydrobacter* was identified as a key biomarker for LPC, whereas *Lautropia* was the most significant one for APC, both with LDA scores greater than 4. Green bars represent taxa enriched in LPC, while red bars indicate taxa more prevalent in APC.

Linear discriminant analysis effect size (LEfSe) was used to identify taxonomic biomarkers that differentiate LPC and APC [37,38]. At the genus level, *Enhydrobacter* showed a remarkably higher abundance in the LPC group (LDA score > 4), while *Lautropia* was significantly more abundant in the APC group (LDA score > 4) (Figure 4, Table S3). These results suggest that *Enhydrobacter* may be associated with a better prognosis, while *Lautropia* may contribute to a more aggressive phenotype.

### 3.5. In Vitro Cell Proliferation Assay

**Figure 5:**
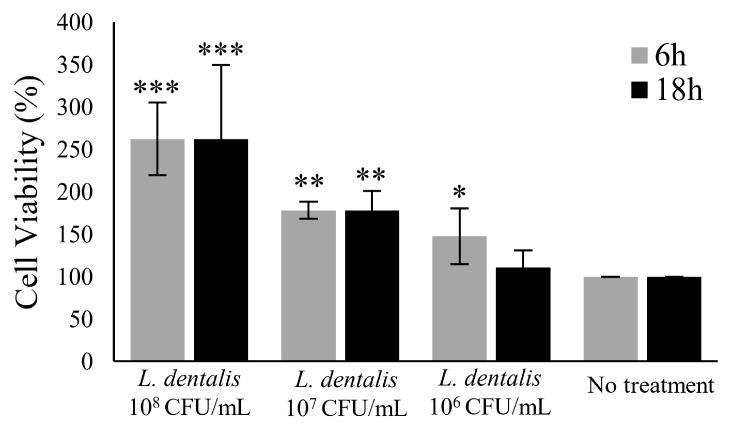
In vitro evaluation of the effect of *Lautropia dentalis* on DU-145 prostate cancer cell viability. The MTT assay was conducted to assess the effect of *Lautropia* strain on DU-145 prostate cancer cells at different bacterial concentrations (10^8^, 10^7^, and 10^6^ CFU/mL) after 6 h and 18 h of exposure. *Lautropia* significantly increased cell viability at all tested concentrations compared to the untreated control. The highest increase in viability was observed at 10^8^ CFU/mL, with a stronger effect at 18 h (*p* < 0.001). A moderate increase was observed at 10^7^ CFU/mL (*p* < 0.01), while 10^6^ CFU/mL showed a more minor but still significant effect (*p* < 0.05). There were no significant changes in the untreated control group. These findings suggest that *Lautropia* enhances the proliferation of DU-145 cells in a manner that is dependent on both time and dose. All data are presented as mean ± standard deviation (SD). Statistical differences between groups were assessed using an unpaired Student’s *t*-test, following confirmation of normality and homogeneity of variances (*** *p* < 0.001; ** *p* < 0.01; * *p* < 0.05).

To assess the functional role of *Lautropia*, an in vitro experiment was performed using DU-145 cells. Cells were treated with various doses (10^6^, 10^7^, 10^8^ CFU/mL) of *Lautropia* for 6 and 18 h. Cell viability was examined by the MTT assay. *Lautropia* exposure resulted in a remarkable dose- and time-dependent increase in cell viability compared to the untreated group (Figure 5). The finding highlights that *Lautropia* may promote prostate cancer cell proliferation.

## 4. Discussion

It is becoming increasingly acknowledged that the gut and intratumoral microbiomes interact intricately with cancer progression. These microbial communities can significantly influence the development and metastasis of cancer [12,13]. Mechanisms include the induction of inflammation and immune system dysregulation, metabolic alterations affecting cancer cell growth and spread, and promotion of angiogenesis via microbial metabolite production [14,15]. This study provides compelling evidence supporting the hypothesis that alterations within the intratumoral microbiome are strongly associated with the progression of prostate cancer.

The prostate’s anatomical location makes it accessible to bacteria that originate in the intestinal and dermatological communities. Therefore, it is possible that the microbes in the prostate originated from one of these populations. *Cutibacterium* spp. are abundant and primarily composed of *C. acnes*, which is consistent with the bacterium’s known role in pro-inflammatory processes. It confirms that *C. acnes* and prostate neoplasia are related, as previously indicated [39,40,41]. Due to their capacity to form biofilms and attach to components of the extracellular matrix (for example, fibronectin), *Corynebacteriaceae* are more commonly associated with prostate diseases, as primarily demonstrated by *Corynebacterium* spp. The probability of tissue invasion is commonly linked to this process [42]. Additionally, these microbes are well-known causative agents of urethral or urinary tract infections [43]. The significant enrichment of Proteobacteria in APC and Bacteroidetes in LPC is consistent with previous reports on microbial community shifts during cancer progression in other tissues. Proteobacteria’s association with inflammation and immune dysregulation has been well-documented in several cancer types [44]. This may be a factor driving cancer progression in APC. Conversely, the higher proportion of Bacteroidetes, often linked to anti-inflammatory effects, in LPC may reflect a different microenvironmental landscape in early-stage disease [45].

Our results reveal a remarkable shift in microbial community composition and richness between localized prostate cancer (LPC) and advanced prostate cancer (APC). The decrease in species richness observed in APC, as measured by ACE analysis, while overall diversity remained stable, suggests a selective pressure within the tumor microenvironment during disease progression. The significant decrease in *Enhydrobacter*, a genus previously associated with positive prognostic indicators in other cancers, and the parallel increase in *Lautropia* suggest that these microbes play a distinct functional role in tumor development and progression [16,17]. The precise mechanisms by which these bacteria exert their influence on prostate cancer warrant further exploration.

The enrichment of *Lautropia* in APC and its ability to increase DU145 cell viability in vitro suggest a possible role in cancer progression. Given that *Lautropia* had the highest LDA score associated with APC cancer, we used DU-145 cells, a traditional cell line frequently used in prostate cancer research, to perform further cell proliferation experiments [46]. When cells were treated with *Lautropia*, a notable increase in cell proliferation was noted. This result was consistent with other research, which has shown a strong correlation between this bacterium and cancer progression. As we aimed to explore the microbial interaction across a broader spectrum of prostate cancer, we used the DU-145 cell line, which has served as a well-established model of advanced and therapy-resistant prostate cancer. Furthermore, DU-145 has been widely used in prior microbiome-related studies assessing tumor cell proliferation, apoptosis, and migration in advanced prostate cancer [47,48]. A study revealed an increased abundance of this bacterium in multiple mucosal organs in cervical cancer patients [49]. These findings of the in vitro assay were consistent with findings from clinical samples, suggesting a link between *Lautropia* and APC.

*Lautropia*, while less studied than other bacterial genera in cancer contexts, is associated with inflammation and disease in other systems. The mechanism by which it promotes prostate cancer cell proliferation requires further investigation, including potential investigation of its secreted metabolites or interactions with host cells. Further experiments, such as functional genomic and metabolomic analyses, will be crucial in clarifying its mode of action, as the current findings remain hypothetical in nature.

Future research should focus on mechanistic investigations into how the identified microbial biomarkers (*Enhydrobacter* and *Lautropia*) interact with host cells and influence tumor progression. This could involve investigations into the production of specific metabolites, their impact on host immune responses, and their contribution to cancer hallmarks such as angiogenesis and metastasis. The development of innovative therapeutic strategies targeting the intratumoral microbiome holds significant promise for improved prostate cancer treatment outcomes. This might involve the use of targeted antimicrobial agents, probiotics, or fecal microbiome transplantation to modulate the composition, thereby influencing tumor development and progression.

The limitations of our study, primarily the relatively small sample size and focus on a single geographic region, are acknowledged. Furthermore, the absence of a proper control group reflects ethical and logistical challenges in obtaining such samples. While we recognize this limitation, we have mitigated it by performing internal comparisons with referenced data from prior studies where applicable. Additionally, for biomarker identification, LEfSe was utilized. Although it has certain limitations, it was used due to its exploratory nature in biomarker discovery, utilizing robust statistical models. Moreover, we acknowledge that specific key pathological parameters—such as lymph node involvement (N stage) and surgical margin status—were not uniformly available across all cases due to the retrospective nature of the study and variability in pathological reporting. As a result, these variables were not included in the analysis, which may limit the completeness of clinicopathological correlation. In addition, the small sample size limited the statistical relevance of key demographic and clinical variables, and these limitations are now acknowledged. Furthermore, while the MTT assay effectively assessed cell viability and proliferation, it does not fully capture other malignant features, such as invasiveness or metastatic potential. Nevertheless, the findings suggest significant correlations between microbial changes and tumor progression. This underscores the need for larger-scale studies with diverse patient cohorts across different geographic locations to strengthen these findings and assess their generalizability. Moreover, longitudinal studies that track changes in microbial communities over time could provide valuable insights into the dynamic interactions between the microbiome and the evolution of prostate cancer.

The integration of microbiome profiling into clinical practice could revolutionize prostate cancer management by enabling more personalized and predictive approaches [50]. As microbial signatures become better characterized, they may serve not only as biomarkers for disease staging but also as indicators of therapeutic responsiveness [51]. For instance, patients with microbiome profiles linked to aggressive disease could benefit from intensified surveillance or adjunctive therapies targeting microbial components. Additionally, understanding how specific bacteria modulate immune responses may inform immunotherapy strategies, potentially enhancing efficacy in resistant cases. Bridging microbiome science with clinical oncology offers a promising frontier for improving patient outcomes and tailoring interventions to individual tumor–microbiome dynamics.

In conclusion, this study provides strong evidence linking alterations in the intratumoral microbiome with prostate cancer progression. While further research is needed to elucidate the complex mechanisms involved, the findings highlight the potential of microbiome-targeted therapies as a novel approach for treating and preventing prostate cancer.

## 5. Conclusions

This study revealed a significant association between alterations in the intratumoral microbiome and prostate cancer progression. Specific microbial taxa, notably *Enhydrobac-ter* and *Lautropia*, showed distinct patterns of abundance in localized versus advanced prostate cancer, potentially influencing metastatic processes. These findings support the potential of microbiome-targeted therapies in the management of prostate cancer. More investigation is warranted.

## Data Availability

The raw sequencing data have been deposited in the Sequence Read Archive (SRA) under BioProject ID PRJNA927108 (https://www.ncbi.nlm.nih.gov/sra/PRJNA927108).

## Supplementary Materials

The following supporting information can be downloaded at https://www.mdpi.com/article/10.3390/biomedicines13081929/s1. Table S1. Clinical and pathological characteristics of the study subjects; Table S2. Averaged taxonomic composition (phylum, class, order, family, genus) in the local prostate cancer (LPC) and advanced prostate cancer (APC) groups; Table S3. Taxonomic biomarker identification in the local prostate cancer (LPC) and advanced prostate cancer (APC) groups.

## Abbreviations

Localized prostate cancer (LPC); advanced prostate cancer (APC); next-generation sequencing (NGS); permutational multivariate analysis of variance (PERMANOVA); principal coordinate analysis (PCoA); unweighted pair group method with arithmetic mean (UPGMA); linear discriminant analysis effect size (LEfSe).
