# Supplementary material for: Association of Intratumoral Microbiota Modulation with Prostate Cancer Progression: A Microbiome Analysis of Prostatic Tissue
Source: Biomedicines. 2025 Aug 7;13(8):1929. doi: 10.3390/biomedicines13081929 (PMC12383511; doi:10.3390/biomedicines13081929)
Supplement: Supplementary file 1 [file biomedicines-13-01929-s001.zip › biomedicines-3763762-supplementary.pdf]

# Supplementary data

**Table S1.** Clinical and pathological characteristics of the study subjects.

| Age | Height | Weight | PSA-Before surgery | Gleason score-after surgery | Grade group | Risk stratification | Pathologic_T_stage |
|---|---|---|---|---|---|---|---|
| 66 | 165 | 68 | 8.17 | 8 | 4 | 3 | 1 |
| 73 | 164 | 48 | 6.55 | 9 | 5 | 3 | 1 |
| 75 | 172 | 60 | 4.7 | 9 | 5 | 4 | 1 |
| 80 | 174 | 63 | 4.8 | 8 | 4 | 4 | 1 |
| 75 | 167 | 65 | 5.3 | 7 | 3 | 2 | 1 |
| 71 | 158 | 50 | 7.5 | 7 | 3 | 2 | 1 |
| 75 | 170 | 65 | 18.8 | 8 | 4 | 3 | 1 |
| 82 | 155 | 54 | 6.6 | 7 | 2 | 2 | 1 |
| 72 | 167 | 60 | 1.9 | 8 | 4 | 3 | 1 |
| 78 | 170 | 57 | 10.7 | 9 | 5 | 3 | 1 |
| 73 | 167 | 64.1 | 9.7 | 7 | 2 | 2 | 2 |
| 65 | 160 | 49 | 4.8 | 7 | 2 | 2 | 2 |
| 75 | 170 | 81.5 | 9.07 | 7 | 3 | 2 | 2 |
| 67 | 174 | 61.2 | 3.8 | 6 | 1 | 1 | 2 |
| 61 | 167 | 77.2 | 7.07 | 7 | 3 | 2 | 2 |
| 75 | 167 | 73 | 4.48 | 7 | 2 | 2 | 2 |
| 64 | 162 | 65.2 | 4.16 | 7 | 2 | 2 | 2 |
| 58 | 172 | 73 | 4.16 | 9 | 5 | 3 | 2 |
| 71 | 160 | 68.6 | 4.94 | 5 | 1 | 1 | 2 |
| 72 | 170 | 62 | 90.5 | 7 | 3 | 3 | 2 |
| 73 | 171 | 63.3 | 7.32 | 6 | 1 | 1 | 2 |
| 75 | 158 | 63 | 20.8 | 9 | 5 | 3 | 3 |
| 76 | 165 | 75 | 8.17 | 7 | 2 | 3 | 3 |
| 72 | 171.6 | 74.1 | 25.98 | 7 | 2 | 3 | 3 |
| 66 | 160.3 | 49.2 | 25.6 | 9 | 5 | 3 | 3 |
| 62 | 165 | 65 | 62.9 | 7 | 2 | 3 | 4 |

PSA, Prostate-specific antigen; T stage, Tumor stage in TNM classification.

**Table S2**. Averaged taxonomic composition (phylum, class, order, family, genus) in the local prostate cancer (LPC) and advanced prostate cancer (APC) groups.

| Taxon Rank | Taxon name | Local prostate cancer (LPC) | | | | | | | Advanced prostate cancer (APC) | | | | | | | p-value |
|---|---|---|---|---|---|---|---|---|---|---|---|---|---|---|---|---|
| | | Aveg | SD | Min | Max | Q1 | Q2 | Q3 | Aveg | SD | Min | Max | Q1 | Q2 | Q3 | |
| Phylum | Proteobacteria | 38.06 | 14.54 | 20.78 | 79.57 | 29.97 | 36.89 | 39.57 | 52.69 | 14.02 | 35.85 | 67.31 | 40.34 | 56.05 | 63.90 | 0.054 |
| | Bacteroidetes | 35.70 | 14.88 | 1.83 | 54.54 | 31.94 | 35.86 | 44.51 | 17.15 | 12.46 | 2.50 | 30.29 | 8.85 | 14.40 | 29.69 | 0.016 |
| | Firmicutes | 20.97 | 4.86 | 8.81 | 27.77 | 20.62 | 22.45 | 23.44 | 20.49 | 7.98 | 9.94 | 27.50 | 14.12 | 23.90 | 26.98 | N.S. |
| | Actinobacteria | 4.87 | 5.26 | 1.58 | 22.99 | 2.11 | 2.88 | 4.85 | 9.19 | 7.87 | 2.14 | 19.39 | 2.64 | 6.13 | 15.64 | N.S. |
| | Unclassified | 0.00 | 0.00 | 0.00 | 0.00 | 0.00 | 0.00 | 0.00 | 0.00 | 0.00 | 0.00 | 0.00 | 0.00 | 0.00 | 0.00 | N.S. |
| | ETC [ < 1.0%] | 0.41 | 0.15 | 0.07 | 0.66 | 0.33 | 0.40 | 0.51 | 0.48 | 0.42 | 0.21 | 1.21 | 0.23 | 0.34 | 0.43 | N.S. |
| Class | Bacteroidia | 35.26 | 15.17 | 0.00 | 54.46 | 31.33 | 35.50 | 44.36 | 16.35 | 12.62 | 1.62 | 29.61 | 7.64 | 13.82 | 29.08 | 0.013 |
| | Gammaproteobacteria | 16.97 | 10.10 | 4.06 | 50.27 | 12.24 | 14.31 | 20.70 | 15.12 | 7.59 | 7.60 | 24.52 | 10.64 | 10.82 | 21.99 | N.S. |
| | Clostridia | 15.52 | 5.89 | 1.04 | 21.52 | 14.74 | 17.07 | 18.95 | 8.99 | 8.16 | 1.57 | 22.58 | 4.00 | 7.64 | 9.18 | N.S. |
| | Betaproteobacteria | 14.79 | 6.29 | 5.91 | 30.18 | 10.71 | 12.07 | 17.63 | 24.62 | 6.93 | 19.42 | 36.78 | 21.63 | 22.17 | 23.10 | 0.010 |
| | Bacilli | 5.27 | 3.18 | 1.59 | 11.65 | 2.68 | 4.59 | 7.65 | 11.25 | 6.11 | 4.20 | 18.21 | 5.80 | 12.27 | 15.79 | 0.037 |
| | Actinobacteria_c | 4.86 | 5.25 | 1.58 | 22.98 | 2.11 | 2.88 | 4.85 | 9.18 | 7.86 | 2.14 | 19.37 | 2.64 | 6.13 | 15.63 | N.S. |
| | Alphaproteobacteria | 1.06 | 3.38 | 2.74 | 14.26 | 4.20 | 5.85 | 7.10 | 1.33 | 7.48 | 6.43 | 22.08 | 7.53 | 8.54 | 19.96 | 0.020 |
| | Flavobacteria | 0.00 | 0.00 | 0.00 | 0.00 | 0.00 | 0.00 | 0.00 | 0.24 | 0.54 | 0.00 | 1.21 | 0.00 | 0.00 | 0.00 | N.S. |
| | Unclassified | 0.00 | 0.00 | 0.00 | 0.00 | 0.00 | 0.00 | 0.00 | 0.00 | 0.00 | 0.00 | 0.00 | 0.00 | 0.00 | 0.00 | N.S. |
| | ETC [ < 1.0%] | 6.28 | 0.60 | 0.34 | 2.73 | 0.58 | 0.96 | 1.39 | 12.91 | 0.29 | 1.01 | 1.68 | 1.15 | 1.20 | 1.60 | N.S. |
| Order | Bacteroidales | 35.26 | 15.17 | 0.00 | 54.46 | 31.33 | 35.50 | 44.36 | 16.35 | 12.62 | 1.62 | 29.61 | 7.64 | 13.82 | 29.08 | 0.013 |
| | Clostridiales | 15.49 | 5.92 | 1.04 | 21.52 | 14.74 | 17.07 | 18.94 | 8.95 | 8.11 | 1.57 | 22.44 | 3.95 | 7.64 | 9.14 | N.S. |
| | Burkholderiales | 14.38 | 6.30 | 5.26 | 29.67 | 10.51 | 11.79 | 17.36 | 23.60 | 6.32 | 17.86 | 34.37 | 20.81 | 22.03 | 22.92 | 0.008 |
| | Rhizobiales | 5.30 | 3.26 | 2.29 | 13.68 | 2.85 | 4.63 | 5.57 | 6.69 | 3.75 | 2.71 | 12.75 | 4.82 | 6.29 | 6.92 | N.S. |
| | Pseudomonadales | 4.54 | 4.43 | 1.83 | 16.98 | 7.90 | 11.67 | 14.35 | 3.65 | 4.25 | 2.29 | 12.87 | 3.13 | 4.15 | 4.95 | N.S. |
| | Lactobacillales | 3.66 | 2.57 | 0.00 | 8.99 | 1.93 | 2.88 | 5.43 | 7.50 | 5.30 | 2.51 | 15.84 | 3.71 | 6.38 | 9.04 | N.S. |
| | Xanthomonadales | 3.20 | 5.89 | 0.00 | 24.54 | 0.00 | 2.19 | 2.88 | 6.20 | 3.01 | 2.22 | 9.75 | 4.27 | 6.58 | 8.20 | 0.031 |
| | Corynebacteriales | 2.24 | 4.40 | 0.00 | 18.10 | 0.00 | 1.21 | 1.96 | 5.50 | 6.56 | 0.00 | 15.95 | 1.33 | 2.37 | 7.87 | N.S. |
| | Enterobacterales | 2.21 | 4.77 | 0.00 | 20.17 | 0.00 | 1.09 | 2.33 | 2.51 | 3.34 | 0.00 | 8.37 | 1.21 | 1.22 | 1.73 | N.S. |
| | Propionibacteriales | 1.42 | 1.27 | 0.00 | 3.22 | 0.00 | 1.50 | 2.89 | 2.52 | 2.41 | 0.00 | 6.38 | 1.42 | 1.80 | 2.97 | N.S. |
| | Bacillales | 1.31 | 1.09 | 0.00 | 3.02 | 0.00 | 1.65 | 2.20 | 3.76 | 2.37 | 1.69 | 6.74 | 2.08 | 2.37 | 5.89 | 0.031 |
| | Micrococcales | 0.22 | 0.63 | 0.00 | 2.02 | 0.00 | 0.00 | 0.00 | 0.27 | 0.61 | 0.00 | 1.37 | 0.00 | 0.00 | 0.00 | N.S. |
| | Pasteurellales | 0.17 | 0.48 | 0.00 | 1.52 | 0.00 | 0.00 | 0.00 | 0.45 | 0.61 | 0.00 | 1.13 | 0.00 | 0.00 | 1.10 | N.S. |
| | Neisseriales | 0.09 | 0.38 | 0.00 | 1.55 | 0.00 | 0.00 | 0.00 | 0.79 | 1.13 | 0.00 | 2.41 | 0.00 | 0.00 | 1.56 | N.S. |
| | Rhodobacterales | 0.00 | 0.00 | 0.00 | 0.00 | 0.00 | 0.00 | 0.00 | 0.48 | 0.67 | 0.00 | 1.37 | 0.00 | 0.00 | 1.05 | N.S. |
| | Sphingomonadales | 0.00 | 0.00 | 0.00 | 0.00 | 0.00 | 0.00 | 0.00 | 2.38 | 3.27 | 0.00 | 6.35 | 0.00 | 0.00 | 5.55 | N.S. |
| | Flavobacteriales | 0.00 | 0.00 | 0.00 | 0.00 | 0.00 | 0.00 | 0.00 | 0.24 | 0.54 | 0.00 | 1.21 | 0.00 | 0.00 | 0.00 | N.S. |
| | Caulobacterales | 0.00 | 0.00 | 0.00 | 0.00 | 0.00 | 0.00 | 0.00 | 0.26 | 0.58 | 0.00 | 1.31 | 0.00 | 0.00 | 0.00 | N.S. |
| | Rhodospirillales | 0.00 | 0.00 | 0.00 | 0.00 | 0.00 | 0.00 | 0.00 | 2.41 | 5.40 | 0.00 | 12.07 | 0.00 | 0.00 | 0.00 | N.S. |
| | Unclassified | 0.00 | 0.00 | 0.00 | 0.00 | 0.00 | 0.00 | 0.00 | 0.00 | 0.00 | 0.00 | 0.00 | 0.00 | 0.00 | 0.00 | N.S. |
| | ETC [ < 1.0%] | 10.49 | 0.85 | 3.06 | 6.20 | 4.07 | 4.29 | 4.74 | 5.48 | 0.67 | 2.66 | 4.43 | 3.46 | 3.65 | 4.05 | 0.045 |
| Family | Muribaculaceae | 30.50 | 13.16 | 0.00 | 46.18 | 27.53 | 32.57 | 38.09 | 13.35 | 10.83 | 1.06 | 26.00 | 4.87 | 12.26 | 22.58 | 0.013 |
| | Comamonadaceae | 13.54 | 6.08 | 4.60 | 28.20 | 9.66 | 11.25 | 16.62 | 19.91 | 9.54 | 7.19 | 33.33 | 16.06 | 20.41 | 22.57 | N.S. |
| | Moraxellaceae | 8.87 | 4.74 | 1.39 | 15.49 | 4.21 | 9.95 | 12.10 | 3.18 | 2.15 | 1.62 | 6.86 | 2.03 | 2.11 | 3.28 | 0.031 |
| | Ruminococcaceae | 8.31 | 3.45 | 0.00 | 12.35 | 7.95 | 8.93 | 10.20 | 5.08 | 4.66 | 1.14 | 12.81 | 2.03 | 3.74 | 5.64 | N.S. |
| | Bradyrhizobiaceae | 4.99 | 3.30 | 1.87 | 13.47 | 2.26 | 4.42 | 5.26 | 6.25 | 4.02 | 1.39 | 12.44 | 4.63 | 6.09 | 6.71 | N.S. |
| | Christensenellaceae | 4.22 | 1.93 | 0.00 | 6.74 | 3.40 | 4.78 | 5.11 | 2.02 | 2.44 | 0.00 | 5.97 | 0.00 | 1.96 | 2.18 | N.S. |
| | Bacteroidaceae | 3.34 | 1.80 | 0.00 | 7.00 | 2.72 | 3.13 | 4.54 | 1.94 | 2.35 | 0.00 | 5.76 | 0.00 | 1.84 | 2.11 | N.S. |
| | Xanthomonadaceae | 3.20 | 5.89 | 0.00 | 24.54 | 0.00 | 2.19 | 2.88 | 6.20 | 3.01 | 2.22 | 9.75 | 4.27 | 6.58 | 8.20 | 0.031 |
| | Lachnospiraceae | 2.73 | 1.17 | 0.00 | 4.17 | 2.64 | 3.07 | 3.36 | 1.56 | 1.28 | 0.00 | 3.53 | 1.07 | 1.47 | 1.72 | N.S. |
| | Streptococcaceae | 2.12 | 1.97 | 0.00 | 7.67 | 1.39 | 1.70 | 2.44 | 5.80 | 5.75 | 0.00 | 14.75 | 1.83 | 5.10 | 7.33 | N.S. |
| | Yersiniaceae | 2.02 | 4.68 | 0.00 | 19.70 | 0.00 | 0.00 | 2.00 | 2.34 | 3.28 | 0.00 | 8.12 | 1.02 | 1.21 | 1.35 | N.S. |
| | Pseudomonadaceae | 1.49 | 2.81 | 0.00 | 11.98 | 0.00 | 1.25 | 1.44 | 1.99 | 2.54 | 0.00 | 6.00 | 0.00 | 1.01 | 2.92 | N.S. |
| | Propionibacteriaceae | 1.41 | 1.27 | 0.00 | 3.22 | 0.00 | 1.50 | 2.89 | 2.50 | 2.41 | 0.00 | 6.38 | 1.42 | 1.76 | 2.93 | N.S. |
| | Prevotellaceae | 0.97 | 1.49 | 0.00 | 5.05 | 0.00 | 0.00 | 1.23 | 0.35 | 0.77 | 0.00 | 1.73 | 0.00 | 0.00 | 0.00 | N.S. |
| | Corynebacteriaceae | 0.98 | 1.64 | 0.00 | 4.62 | 0.00 | 0.00 | 1.26 | 2.11 | 2.68 | 0.00 | 6.55 | 0.00 | 1.88 | 2.09 | N.S. |
| | Staphylococcaceae | 0.95 | 0.96 | 0.00 | 2.62 | 0.00 | 1.43 | 1.73 | 2.85 | 2.30 | 1.05 | 5.65 | 1.20 | 1.29 | 5.07 | N.S. |
| | Lawsonella_f | 0.86 | 3.24 | 0.00 | 13.39 | 0.00 | 0.00 | 0.00 | 0.00 | 0.00 | 0.00 | 0.00 | 0.00 | 0.00 | 0.00 | N.S. |
| | Lactobacillaceae | 0.31 | 0.74 | 0.00 | 2.54 | 0.00 | 0.00 | 0.00 | 0.00 | 0.00 | 0.00 | 0.00 | 0.00 | 0.00 | 0.00 | N.S. |
| | Pasteurellaceae | 0.17 | 0.48 | 0.00 | 1.52 | 0.00 | 0.00 | 0.00 | 0.45 | 0.61 | 0.00 | 1.13 | 0.00 | 0.00 | 1.10 | N.S. |
| | Leuconostocaceae | 0.13 | 0.36 | 0.00 | 1.09 | 0.00 | 0.00 | 0.00 | 0.00 | 0.00 | 0.00 | 0.00 | 0.00 | 0.00 | 0.00 | N.S. |
| | Neisseriaceae | 0.09 | 0.38 | 0.00 | 1.55 | 0.00 | 0.00 | 0.00 | 0.79 | 1.13 | 0.00 | 2.41 | 0.00 | 0.00 | 1.56 | N.S. |
| | Ralstonia_f | 0.07 | 0.27 | 0.00 | 1.11 | 0.00 | 0.00 | 0.00 | 0.77 | 0.72 | 0.00 | 1.48 | 0.00 | 1.02 | 1.35 | N.S. |
| | Rhodobacteraceae | 0.00 | 0.00 | 0.00 | 0.00 | 0.00 | 0.00 | 0.00 | 0.48 | 0.67 | 0.00 | 1.37 | 0.00 | 0.00 | 1.05 | N.S. |
| | Aerococcaceae | 0.00 | 0.00 | 0.00 | 0.00 | 0.00 | 0.00 | 0.00 | 0.21 | 0.46 | 0.00 | 1.04 | 0.00 | 0.00 | 0.00 | N.S. |
| | Flavobacteriaceae | 0.00 | 0.00 | 0.00 | 0.00 | 0.00 | 0.00 | 0.00 | 0.24 | 0.54 | 0.00 | 1.21 | 0.00 | 0.00 | 0.00 | N.S. |
| | Sphingomonadaceae | 0.00 | 0.00 | 0.00 | 0.00 | 0.00 | 0.00 | 0.00 | 2.37 | 3.26 | 0.00 | 6.32 | 0.00 | 0.00 | 5.53 | N.S. |
| | Lautropia_f | 0.00 | 0.00 | 0.00 | 0.00 | 0.00 | 0.00 | 0.00 | 2.41 | 5.38 | 0.00 | 12.04 | 0.00 | 0.00 | 0.00 | N.S. |
| | Rhodospirillaceae | 0.00 | 0.00 | 0.00 | 0.00 | 0.00 | 0.00 | 0.00 | 2.37 | 5.29 | 0.00 | 11.83 | 0.00 | 0.00 | 0.00 | N.S. |
| | Caulobacteraceae | 0.00 | 0.00 | 0.00 | 0.00 | 0.00 | 0.00 | 0.00 | 0.26 | 0.58 | 0.00 | 1.31 | 0.00 | 0.00 | 0.00 | N.S. |
| | Mycobacteriaceae | 0.00 | 0.00 | 0.00 | 0.00 | 0.00 | 0.00 | 0.00 | 2.73 | 6.10 | 0.00 | 13.64 | 0.00 | 0.00 | 0.00 | N.S. |
| | Unclassified | 0.00 | 0.00 | 0.00 | 0.00 | 0.00 | 0.00 | 0.00 | 0.00 | 0.00 | 0.00 | 0.00 | 0.00 | 0.00 | 0.00 | N.S. |
| | ETC [ < 1.0%] | 8.72 | 2.02 | 5.95 | 13.15 | 7.01 | 8.23 | 9.89 | 9.23 | 2.22 | 6.44 | 12.42 | 8.01 | 9.51 | 9.75 | N.S. |
| Genus | PAC000186_g | 16.43 | 7.24 | 0.00 | 28.81 | 14.87 | 16.85 | 18.84 | 6.83 | 6.25 | 0.00 | 14.79 | 3.08 | 4.34 | 11.93 | 0.013 |
| | Pelomonas | 13.28 | 6.04 | 4.50 | 28.03 | 9.26 | 11.02 | 16.11 | 16.91 | 10.61 | 6.70 | 33.07 | 8.43 | 15.95 | 20.41 | N.S. |
| | Enhydrobacter | 8.10 | 4.96 | 0.00 | 14.82 | 3.35 | 9.70 | 11.17 | 2.20 | 1.77 | 1.02 | 5.26 | 1.15 | 1.42 | 2.17 | 0.031 |
| | Pseudoflavonifractor | 4.22 | 1.78 | 0.00 | 6.86 | 4.06 | 4.74 | 4.92 | 2.61 | 3.47 | 0.00 | 8.41 | 0.00 | 1.72 | 2.93 | N.S. |
| | PAC001066_g | 4.19 | 1.85 | 0.00 | 7.41 | 4.04 | 4.72 | 5.00 | 1.86 | 2.06 | 0.00 | 4.21 | 0.00 | 1.21 | 3.86 | 0.031 |
| | Bradyrhizobium | 3.89 | 2.55 | 1.52 | 10.74 | 1.85 | 3.21 | 4.13 | 4.91 | 2.91 | 1.07 | 8.97 | 3.61 | 4.93 | 5.97 | N.S. |
| | Bacteroides | 3.34 | 1.80 | 0.00 | 7.00 | 2.72 | 3.13 | 4.54 | 1.94 | 2.35 | 0.00 | 5.76 | 0.00 | 1.84 | 2.11 | N.S. |
| | Stenotrophomonas | 2.99 | 5.90 | 0.00 | 24.43 | 0.00 | 1.33 | 2.70 | 4.79 | 4.01 | 0.00 | 9.47 | 2.03 | 4.27 | 8.19 | N.S. |
| | PAC001127_g | 2.85 | 1.23 | 0.00 | 4.76 | 2.71 | 3.17 | 3.36 | 1.03 | 1.42 | 0.00 | 2.85 | 0.00 | 0.00 | 2.29 | 0.016 |
| | Oscillibacter | 2.65 | 1.23 | 0.00 | 3.85 | 2.13 | 3.13 | 3.60 | 0.71 | 1.01 | 0.00 | 2.17 | 0.00 | 0.00 | 1.39 | 0.013 |
| | PAC001068_g | 2.48 | 3.39 | 0.00 | 11.58 | 0.00 | 1.42 | 2.34 | 1.24 | 1.37 | 0.00 | 3.33 | 0.00 | 1.42 | 1.47 | N.S. |
| | Streptococcus | 2.09 | 1.94 | 0.00 | 7.64 | 1.38 | 1.68 | 2.42 | 5.78 | 5.76 | 0.00 | 14.75 | 1.76 | 5.08 | 7.33 | N.S. |
| | Serratia | 1.99 | 4.66 | 0.00 | 19.60 | 0.00 | 0.00 | 1.98 | 2.13 | 3.40 | 0.00 | 8.11 | 0.00 | 1.17 | 1.35 | N.S. |
| | PAC001360_g | 1.70 | 0.97 | 0.00 | 3.08 | 1.10 | 2.10 | 2.29 | 0.39 | 0.88 | 0.00 | 1.97 | 0.00 | 0.00 | 0.00 | 0.025 |
| | Cutibacterium | 1.39 | 1.25 | 0.00 | 3.18 | 0.00 | 1.48 | 2.81 | 2.48 | 2.40 | 0.00 | 6.34 | 1.42 | 1.70 | 2.93 | N.S. |
| | Pseudomonas | 1.37 | 2.78 | 0.00 | 11.78 | 0.00 | 1.17 | 1.38 | 1.78 | 2.68 | 0.00 | 6.00 | 0.00 | 0.00 | 2.92 | N.S. |
| | PAC001063_g | 1.37 | 1.19 | 0.00 | 3.75 | 0.00 | 1.17 | 2.16 | 0.31 | 0.69 | 0.00 | 1.54 | 0.00 | 0.00 | 0.00 | N.S. |
| | PAC001074_g | 1.12 | 0.67 | 0.00 | 1.88 | 1.09 | 1.35 | 1.54 | 0.23 | 0.52 | 0.00 | 1.15 | 0.00 | 0.00 | 0.00 | 0.020 |
| | PAC000664_g | 1.09 | 0.76 | 0.00 | 1.98 | 0.00 | 1.45 | 1.57 | 0.23 | 0.52 | 0.00 | 1.15 | 0.00 | 0.00 | 0.00 | 0.037 |
| | Corynebacterium | 0.97 | 1.62 | 0.00 | 4.54 | 0.00 | 0.00 | 1.22 | 2.05 | 2.60 | 0.00 | 6.35 | 0.00 | 1.81 | 2.09 | N.S. |
| | Prevotella | 0.96 | 1.48 | 0.00 | 5.05 | 0.00 | 0.00 | 1.23 | 0.33 | 0.74 | 0.00 | 1.66 | 0.00 | 0.00 | 0.00 | N.S. |
| | Christensenellaceae_uc | 0.94 | 0.76 | 0.00 | 2.01 | 0.00 | 1.25 | 1.44 | 0.45 | 1.00 | 0.00 | 2.24 | 0.00 | 0.00 | 0.00 | N.S. |
| | Lawsonella | 0.85 | 3.23 | 0.00 | 13.35 | 0.00 | 0.00 | 0.00 | 0.00 | 0.00 | 0.00 | 0.00 | 0.00 | 0.00 | 0.00 | N.S. |
| | Afipia | 0.80 | 0.98 | 0.00 | 2.75 | 0.00 | 0.00 | 1.32 | 1.13 | 1.42 | 0.00 | 3.47 | 0.00 | 1.02 | 1.16 | N.S. |
| | Staphylococcus | 0.73 | 0.87 | 0.00 | 2.50 | 0.00 | 0.00 | 1.26 | 2.32 | 2.76 | 0.00 | 5.58 | 0.00 | 1.02 | 5.03 | N.S. |
| | Lactobacillus | 0.20 | 0.45 | 0.00 | 1.27 | 0.00 | 0.00 | 0.00 | 0.00 | 0.00 | 0.00 | 0.00 | 0.00 | 0.00 | 0.00 | N.S. |
| | PAC001062_g | 0.19 | 0.54 | 0.00 | 1.86 | 0.00 | 0.00 | 0.00 | 0.00 | 0.00 | 0.00 | 0.00 | 0.00 | 0.00 | 0.00 | N.S. |
| | Acinetobacter | 0.15 | 0.43 | 0.00 | 1.36 | 0.00 | 0.00 | 0.00 | 0.00 | 0.00 | 0.00 | 0.00 | 0.00 | 0.00 | 0.00 | N.S. |
| | Muribaculum | 0.15 | 0.44 | 0.00 | 1.34 | 0.00 | 0.00 | 0.00 | 0.00 | 0.00 | 0.00 | 0.00 | 0.00 | 0.00 | 0.00 | N.S. |
| | PAC002400_g | 0.09 | 0.39 | 0.00 | 1.59 | 0.00 | 0.00 | 0.00 | 0.00 | 0.00 | 0.00 | 0.00 | 0.00 | 0.00 | 0.00 | N.S. |
| | Psychrobacter | 0.09 | 0.35 | 0.00 | 1.45 | 0.00 | 0.00 | 0.00 | 0.00 | 0.00 | 0.00 | 0.00 | 0.00 | 0.00 | 0.00 | N.S. |
| | Xanthomonas | 0.09 | 0.36 | 0.00 | 1.48 | 0.00 | 0.00 | 0.00 | 0.00 | 0.00 | 0.00 | 0.00 | 0.00 | 0.00 | 0.00 | N.S. |
| | Neisseria | 0.08 | 0.34 | 0.00 | 1.39 | 0.00 | 0.00 | 0.00 | 0.39 | 0.88 | 0.00 | 1.97 | 0.00 | 0.00 | 0.00 | N.S. |
| | PAC000198_g | 0.07 | 0.27 | 0.00 | 1.12 | 0.00 | 0.00 | 0.00 | 0.00 | 0.00 | 0.00 | 0.00 | 0.00 | 0.00 | 0.00 | N.S. |
| | Haemophilus | 0.07 | 0.28 | 0.00 | 1.15 | 0.00 | 0.00 | 0.00 | 0.00 | 0.00 | 0.00 | 0.00 | 0.00 | 0.00 | 0.00 | N.S. |
| | Pediococcus | 0.07 | 0.31 | 0.00 | 1.26 | 0.00 | 0.00 | 0.00 | 0.00 | 0.00 | 0.00 | 0.00 | 0.00 | 0.00 | 0.00 | N.S. |
| | Ralstonia | 0.06 | 0.26 | 0.00 | 1.09 | 0.00 | 0.00 | 0.00 | 0.76 | 0.72 | 0.00 | 1.48 | 0.00 | 1.02 | 1.32 | N.S. |
| | Sphingomonas | 0.00 | 0.00 | 0.00 | 0.00 | 0.00 | 0.00 | 0.00 | 2.27 | 3.13 | 0.00 | 6.20 | 0.00 | 0.00 | 5.15 | N.S. |
| | Lautropia | 0.00 | 0.00 | 0.00 | 0.00 | 0.00 | 0.00 | 0.00 | 2.27 | 5.08 | 0.00 | 11.36 | 0.00 | 0.00 | 0.00 | N.S. |
| | Mycobacterium | 0.00 | 0.00 | 0.00 | 0.00 | 0.00 | 0.00 | 0.00 | 2.69 | 6.01 | 0.00 | 13.43 | 0.00 | 0.00 | 0.00 | N.S. |
| | JZUE_g | 0.00 | 0.00 | 0.00 | 0.00 | 0.00 | 0.00 | 0.00 | 2.73 | 6.10 | 0.00 | 13.64 | 0.00 | 0.00 | 0.00 | N.S. |
| | Leuconostoc | 0.00 | 0.00 | 0.00 | 0.00 | 0.00 | 0.00 | 0.00 | 0.22 | 0.50 | 0.00 | 1.11 | 0.00 | 0.00 | 0.00 | N.S. |
| | Brevundimonas | 0.00 | 0.00 | 0.00 | 0.00 | 0.00 | 0.00 | 0.00 | 0.25 | 0.57 | 0.00 | 1.27 | 0.00 | 0.00 | 0.00 | N.S. |
| | Chiayiivirga | 0.00 | 0.00 | 0.00 | 0.00 | 0.00 | 0.00 | 0.00 | 0.41 | 0.91 | 0.00 | 2.04 | 0.00 | 0.00 | 0.00 | N.S. |
| | Skermanella | 0.00 | 0.00 | 0.00 | 0.00 | 0.00 | 0.00 | 0.00 | 2.29 | 5.12 | 0.00 | 11.45 | 0.00 | 0.00 | 0.00 | N.S. |
| | Luteimonas | 0.00 | 0.00 | 0.00 | 0.00 | 0.00 | 0.00 | 0.00 | 0.45 | 1.00 | 0.00 | 2.24 | 0.00 | 0.00 | 0.00 | N.S. |
| | Unclassified | 0.02 | 0.04 | 0.00 | 0.14 | 0.00 | 0.00 | 0.02 | 0.00 | 0.01 | 0.00 | 0.01 | 0.00 | 0.00 | 0.01 | N.S. |
| | ETC [ < 1.0%] | 16.86 | 2.28 | 13.85 | 21.89 | 15.04 | 16.81 | 18.40 | 20.60 | 4.05 | 17.15 | 27.25 | 17.60 | 19.98 | 21.01 | 0.025 |

Relative abundance less than 1% was expressed as Et cetera (ETC). The Wilcoxon rank sum test was used to analyze the significance between the two groups. *, $p < 0.05$ and N.S. Not significant.

**Table S3.** Taxonomic biomarker identification in the local prostate cancer (LPC) and advanced prostate cancer (APC) group.

| Taxon name | Taxon rank | LDA effect size | *p*-value | LPC | APC |
|---|---|---|---|---|---|
| Bacteroidetes | Phylum | 4.9 | 0.017 | 35.5 | 17.2 |
| Bacteroidia | Class | 4.9 | 0.014 | 35.1 | 16.5 |
| Betaproteobacteria | Class | 4.7 | 0.000 | 14.9 | 24.9 |
| Bacteroidales | Order | 4.9 | 0.014 | 35.1 | 16.5 |
| Caulobacterales | Order | 3.4 | 0.026 | 0.1 | 0.6 |
| Bacilli | Class | 4.5 | 0.046 | 5.3 | 11.0 |
| Alphaproteobacteria | Class | 4.5 | 0.021 | 6.6 | 13.1 |
| Burkholderiales | Order | 4.6 | 0.009 | 14.4 | 24.1 |
| Pseudomonadales | Order | 4.4 | 0.026 | 10.2 | 5.2 |
| Xanthomonadales | Order | 4.1 | 0.021 | 3.3 | 6.0 |
| Bacillales | Order | 4.0 | 0.041 | 1.4 | 3.7 |
| Muribaculaceae | Family | 4.9 | 0.014 | 30.3 | 13.3 |
| Moraxellaceae | Family | 4.4 | 0.015 | 8.6 | 3.0 |
| Lautropia_f | Family | 4.2 | 0.006 | 0.0 | 2.6 |
| Xanthomonadaceae | Family | 4.1 | 0.021 | 3.3 | 6.0 |
| Sphingomonadaceae | Family | 4.0 | 0.044 | 0.4 | 2.6 |
| Caulobacteraceae | Family | 3.4 | 0.026 | 0.1 | 0.6 |
| Gemella_f | Family | 3.3 | 0.010 | 0.1 | 0.5 |
| Methylobacteriaceae | Family | 3.0 | 0.021 | 0.2 | 0.0 |
| *Enhydrobacter* | Genus | 4.4 | 0.014 | 7.8 | 1.9 |
| *Lautropia* | Genus | 4.2 | 0.006 | 0.0 | 2.4 |
| *Sphingomonas* | Genus | 4.0 | 0.044 | 0.3 | 2.6 |
| *Oscillibacter* | Genus | 3.8 | 0.023 | 2.7 | 1.2 |
| *Gemella* | Genus | 3.3 | 0.010 | 0.1 | 0.5 |
| *Brevundimonas* | Genus | 3.3 | 0.026 | 0.1 | 0.5 |
| *Luteimonas* | Genus | 3.2 | 0.001 | 0.0 | 0.4 |
